# Visualizing the endothelial glycocalyx in human glioma vasculature

**DOI:** 10.1007/s10014-025-00498-z

**Published:** 2025-03-04

**Authors:** Kazufumi Ohmura, Hiroyuki Tomita, Hideshi Okada, Noriyuki Nakayama, Naoyuki Ohe, Tsuyoshi Izumo, Akira Hara

**Affiliations:** 1 Department of Neurosurgery, Gifu University Graduate School of Medicine, Gifu, Japan; 2 Department of Tumor Pathology, Gifu University Graduate School of Medicine, 1-1 Yanagido, Gifu, 501-1194 Japan; 3 Center for One Medicine Innovative Translational Research, Gifu University Institute for Advanced Study, Gifu, Japan; 4 Department of Emergency and Disaster Medicine, Gifu University Graduate School of Medicine, 1-1 Yanagido, Gifu, 501-1194 Japan

**Keywords:** Glioma, Endothelial glycocalyx, Blood–brain barrier, Blood–tumor barrier, Glioblastoma, Electron microscopy

## Abstract

Gliomas are the most common primary brain tumors in adults. However, glioblastoma is especially difficult to treat despite advancements in treatment. Therefore, new and more effective treatments are needed. The endothelial glycocalyx covers the luminal surface of the endothelium and plays an important role in vascular homeostasis. Tumor blood vessels normally have increased permeability, but some of them mimic normal cerebral blood vessels constituting the blood–brain barrier and retain drug-barrier function. Therefore, brain tumor vessels are considered to constitute the blood–tumor barrier. There are few reports on the endothelial glycocalyx in human brain tumor vessels. We aimed to visualize the endothelial glycocalyx in human brain tumor vessels and evaluate its microstructural differences in glioma vessels and normal capillaries. Surgical specimens from patients with glioma who underwent tumor resection at our institution were evaluated. We visualized the microstructures of the brain tumor vessels in human glioma specimens using electron microscopy with lanthanum nitrate. The endothelial glycocalyx was identified in the human glioma vasculature and its microstructure varied between the tumor margin and core. These variations may influence tumor angiogenesis and vascular remodeling, contributing to advancements in targeted therapies and diagnostics for human gliomas.

**Supplementary Information:**

The online version contains supplementary material available at 10.1007/s10014-025-00498-z.

## Introduction

Gliomas are the most common primary brain tumors in adults. Glioblastoma has a poor prognosis and is incurable despite several available therapies, including surgery, radiotherapy, and chemotherapy. Glioblastoma is characterized by a strong invasive potential in the normal brain and angiogenesis [1]. Various approaches have been used to control glioma invasion and angiogenesis [2]. Bevacizumab, a monoclonal antibody against vascular endothelial growth factor A that promotes angiogenesis, has been used to control angiogenesis in recurrent glioblastoma. Bevacizumab has the clinical benefits of reducing peritumoral edema and steroid dependence, but it does not improve the overall survival in patients with glioblastoma [3]. Therefore, there is an urgent need to develop new approaches that target the vascular endothelium beyond merely controlling angiogenesis to improve patient prognosis [4].

Normal cerebral capillaries act as the blood–brain barrier (BBB) and prevent harmful substances from entering the brain. In contrast, tumor blood vessels have been suggested to protect brain tumors by acting as a blood–tumor barrier [4, 5]. Tumor vessels retain a drug-barrier function similar to normal cerebral capillaries and protect brain tumors through heterogeneous drug permeability and aggressive efflux of molecules [5]. These vessels are clinically visible as a gadolinium-enhanced area on magnetic resonance imaging (MRI) or as a dense network of vessels on angiography.

Neuropathologists have provided remarkable insights into the vascular microstructure of gliomas. Glioma vessels have larger diameters than normal cerebral vessels with irregular luminal surfaces, and their intraluminal flap extends into the lumen [6–8]. Some glioma tumor vessels have been reported to mimic normal cerebral vessels and may have features common to cerebral capillaries, such as retained tight junctions and lack of fenestration [7]. However, reports on the endothelial glycocalyx in human gliomas are lacking.

The endothelial glycocalyx is a generic term for polysaccharides and glycoproteins on the surface of endothelial cells that maintain microcirculatory homeostasis by regulating microvascular tonus, vascular permeability, leukocyte migration, and intravascular thrombi [9]. Vascular permeability, which refers to the ability of fluids such as water, oxygen, and nutrients to move between capillaries and tissues, depends on the endothelial glycocalyx [10]. Our group reported that the endothelial glycocalyx in mouse brain capillaries was markedly thicker than those in other organs [11]. This suggests that the endothelial glycocalyx plays an important role in protecting the brain at the forefront of the endothelium and acts as a BBB.

The endothelial glycocalyx is also involved in processes underlying cancer progression, including tumor cell adhesion, tumor formation, and tumor growth [12, 13]. Sialic acid metabolism, a component of the glycocalyx, affects glioblastoma growth and network formation [14]. Apart from a study reporting that a decrease in the concentration of heparan sulfate, the main component of the endothelial glycocalyx, suppressed the growth of glioblastoma in a mouse model [15], few studies on the endothelial glycocalyx in human glioma have been published.

The morphology and properties of the glycocalyx covering the cell surface of the endothelium and tumors have mostly been reported in animal models, such as mice and rats, and cultured human cells [16]. However, these results did not provide the true appearance of the glycocalyx in humans [17].

This study aimed to visualize the human glioma endothelial glycocalyx using surgical specimens and evaluate the microstructural differences in the endothelial glycocalyx of human gliomas in glioma vessels and normal capillaries.

## Methods

### Patients

Nine patients with glioma who underwent tumor resection at Gifu University Hospital between June 30, 2023 and May 10, 2024 were included in the study. They were pathologically diagnosed, and their tumors were classified according to the 2021 WHO classification. All patients provided consent. The study was approved by the Ethics Committee of Gifu University Graduate School of Medicine (Number: 2023-025).

### Surgery

All patients underwent maximum tumor resection performed by N.N. using an exoscope (ORBEYE; Olympus, Tokyo, Japan) and a navigation system (BrainLAB, Munich, Germany). Gadolinium-enhanced MRI (Gd-MRI) was used as the baseline for navigation systems.

### MRI

Three-dimensional turbo field echo T1-weighted images were acquired under the following conditions: repetition time, 15 ms; echo time, 3.8 ms; field of view, 26 × 26 cm; matrix size, 288 × 288 in the sagittal plane using a 3 T MRI machine (Intera Achieva Quasar-dual 3.0; Philips, Best, the Netherlands). Transaxial images were obtained from the sagittal images. The slice thickness was 3 mm, and the slice gap was 1 mm. Gadobutrol (Gadovist; Bayer, Leverkusen, Germany), a gadolinium-based contrast agent, was administered at 0.1 mmol/kg for the contrast sequence. The tumor vessels in the gadolinium-enhanced area were initially observed using an optical microscope. This was followed by assessment of the MRI and histopathological images of the same sites.

### Tissue preparation

Surgical specimens were collected immediately after tumor resection to prepare frozen sections. Each sample consisted of approximately 5-mm^3^ cubes of tissue. Tissues were protected with Tissue OCT compound (Sakura Finetek, Japan), snap-frozen in liquid nitrogen, and stored at − 80 °C. Next, 5-μm serial sections were generated using a Leica CM1850 cryostat (Leica Microsystems, Wetzlar, Germany). The sections were air-dried at room temperature for 30 min. For the preparation of the formalin-fixed paraffin-embedded sections, the surgical specimens were fixed in 10% neutral buffered formalin, routinely processed, and whole-mount embedded.

### Histological and immunohistochemical procedures for paraffin sections

Paraffin blocks were dissected into 3-μm thick sections and subjected to hematoxylin and eosin (HE) staining using a routine procedure. Adjacent serial sections were subjected to immunohistochemistry for CD31. For immunostaining, deparaffinized sections were subjected to autoclave boiling in Tris EDTA buffer solution (pH 9.0) for 10 min at 110 °C as an antigen retrieval procedure before incubation with 3% H_2_O_2_ diluted in methanol for 10 min and blocked with 2% normal bovine serum. The sections were incubated with rabbit anti-CD31 antibody (dilution 1:200, ab76533; Abcam, Cambridge, UK) overnight at 4 °C, followed by incubation with peroxidase-labeled anti-rabbit antibody (Histofine Simplestain Max PO [R]; Nichirei) for 60 min at 37 °C. The immunoreaction was visualized using 3,3′-diaminobenzidine tetrahydrochloride (DAB; Sigma-Aldrich, St. Louis, MO, USA). The sections were counterstained with hematoxylin, mounted on slides with mounting medium, and topped with coverslips.

### Double staining with fluorescence-labeled lectins and anti-CD31 antibody

Twenty different types of lectins (lectin screening kits; I–II, Vector Laboratories, Newark, CA) were used in this study. Lectins are useful in detecting glycan expression in tissue sections and are classified into five groups according to their binding specificity and inhibitory sugars: *N*-acetylglucosamine, mannose, *N*-acetylgalactosamine, complex-type *N*-glycan groups, and fucose [18]. The fresh frozen sections were fixed for 15 min in 4% paraformaldehyde (0.1 M phosphate buffered saline [PBS], pH 7.4). They were subsequently soaked in PBS for 10 min, placed in carbon-free blocking solution (CFBS; Vector Laboratories), and maintained at room temperature for 60 min. Next, a mixture of biotinylated-lectin and a rabbit monoclonal anti-CD 31 antibody (dilution 1:200, ab76533; Abcam) was added and kept overnight in a refrigerator at 4 °C. After soaking in PBS for 15 min the next day, streptavidin-DyLight 594 (dilution 1:200, Vector Laboratories) and goat anti-rabbit IgG H&L-DyLight 488 (dilution 1:200, ab96899; Abcam) were added, and sections were maintained at room temperature for 60 min. They were soaked in PBS for 15 min, followed by 4ʹ,6-diamidino-2-phenylindole dihydrochloride (DAPI; DOJINDO, Kumamoto, Japan) for 5 min. The sections were soaked again in PBS for 5 min, sealed with a fluorescent anti-extinguishing sealant, and observed under a microscope (BX53; Olympus) [19].

### Assessment of lectin-staining intensity

ImageJ software was used for the quantitative analysis of fluorescence intensity. The intensity analysis was performed manually with 40 high-power fields per sample (*n* = 3 per sample) in the focal plane. A straight line was drawn on the merged image, with green representing the endothelium and red indicating the presence of lectins. The staining intensity profile of lectin was represented by a line on the corresponding image [20]. The areas where the lectin intensity profile (red channel) showed higher values than the CD31 intensity profile (green channel) were identified in the curve graph of the 20 different types of lectin staining.

### Scanning and transmission electron microscopy

Sample preparation for electron microscopy was performed as described previously [21, 22]. The tissue samples were diced into approximately 5-mm^3^ cubes for scanning electron microscopy (SEM) and 1-mm^3^ cubes for transmission electron microscopy (TEM). Some of these tissue pieces were light-shielded and soaked overnight in 2% glutaraldehyde at 4 °C. To observe the microstructure of the endothelial glycocalyx using electron microscopy, the rest of the tissue pieces were immersed overnight in a solution comprising 2% glutaraldehyde, 2% sucrose, 0.1 M sodium cacodylate buffer (pH 7.3), and 2% lanthanum nitrate. They were soaked overnight in a solution without glutaraldehyde the next day and washed in an alkaline (0.03 M NaOH) sucrose (2%) solution. The specimens were dehydrated using a graded ethanol series. The frozen fracture method was used to prepare the samples for 3D examination using SEM. Each sample was placed on an iron plate chilled with liquid nitrogen, and ethanol was sprinkled onto it. The sample was fractured using a chisel once the ethanol was frozen, such that it was not directly touched. They were incubated with tert-butyl alcohol at room temperature. The tert-butyl alcohol was freeze-dried after it solidified, and the specimens were examined using SEM (S-4800; Hitachi, Tokyo, Japan). To prepare the samples for TEM, each specimen was embedded in an epoxy resin. Ultrathin sections (90 nm) stained with uranyl acetate and lead citrate were examined using TEM (HT-7800; Hitachi).

### Quantitative assessments of the endothelial glycocalyx height

Quantitative morphometric analyses were conducted on coded samples by an examiner blinded to the case details. The endothelial glycocalyx height was assessed by selecting five capillary vessels randomly chosen from TEM images for each case. The average height of the endothelial glycocalyx was determined by taking measurements at five points along the vessel wall, except for the nuclear part [21].

### Statistical analysis

For the assessment of lectin-staining intensity, statistical significance was determined using the Wilcoxon rank-sum test. All statistical analyses were performed using JMP 14.2 software (SAS Institute, Cary, NC, USA). Statistical significance was set at *p* < 0.05 for all analyses. All data are presented as mean ± standard error of the mean.

## Results

### Tumor vessels of gliomas are observed in GD-MRI region

The patient characteristics are presented in Table S1. Nine patients were included, and their ages ranged from 26 to 82 years. Five patients had glioblastoma, IDH-wildtype (glioblastoma); three had astrocytoma, IDH-mutant, grade 4 (astrocytoma); and one had oligodendroglioma, IDH-mutant and 1p/19q-codeleted, grade 3 (oligodendroglioma). Of the nine cases, six were primary and three were recurrent. All patients underwent preoperative Gd-MRI. All gliomas showed gadolinium-enhanced areas. The tumor vessels were clinically highlighted as gadolinium-contrast areas and observed in three types of gliomas: glioblastoma, astrocytoma, and oligodendroglioma (Fig. S1). The tumor vessels of the gliomas were commonly positive for CD31, an endothelial marker.

### Endothelial glycocalyx is visualized in tumor vessels of gliomas using electron microscopy

**Fig. 1:**
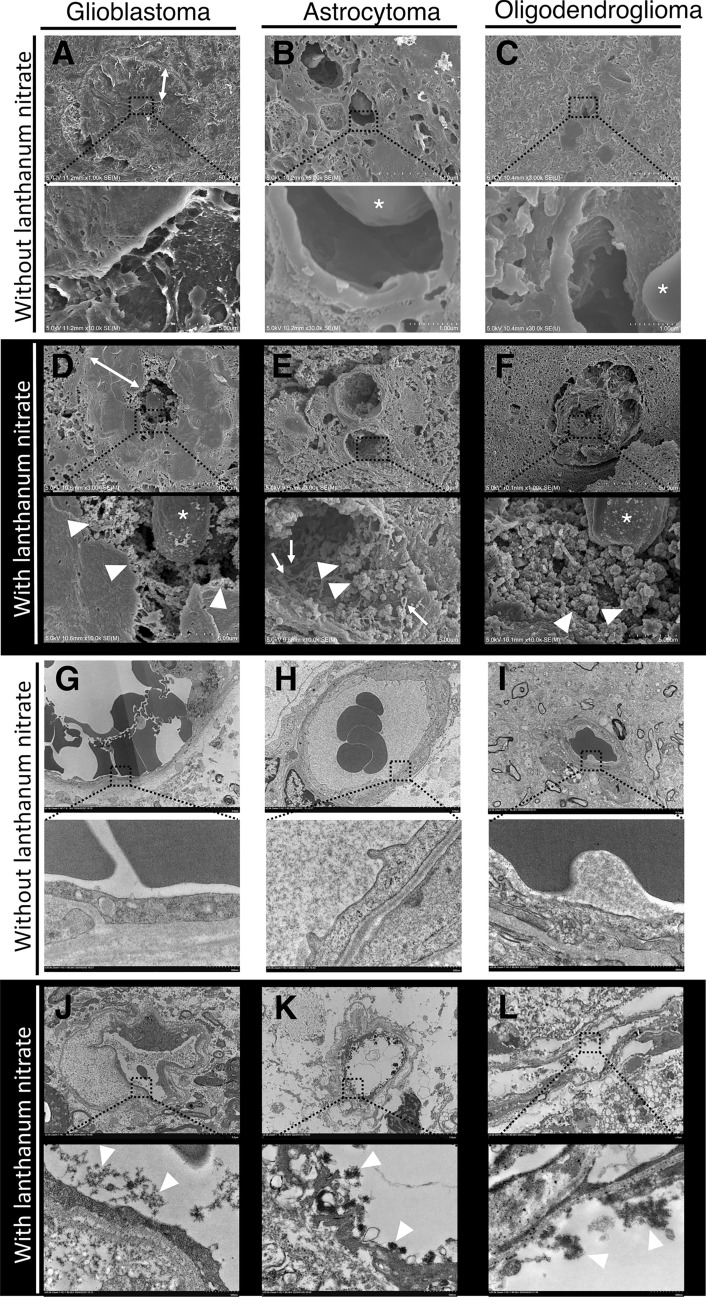
Endothelial glycocalyx in human glioma vessels is visualized using SEM and TEM. Representative SEM (**a–f**) and TEM (**g–l**) images of gliomas: (**a, d, g, j**) glioblastoma, IDH-wildtype (case 3); (**b, e, h, k**) astrocytoma, IDH-mutant, grade 4 (case 2); and (**c, f, i, l**) oligodendroglioma, IDH-mutant and 1p/19q-codeleted, grade 3 (case 6). (**a–c**) and (**g–i**) are observed without lanthanum nitrate to visualize the lumen of the vessels without the glycocalyx. (**d–f**) and (**j–l**) show lanthanum nitrate electron staining for visualization of the endothelial glycocalyx. (**a**) Glioblastoma shows a thickened vessel wall (arrow). No endothelial glycocalyx is observed in (**a**); however, it is observed using lanthanum nitrate in (**d**) (arrowheads). The asterisks indicate erythrocytes. In the astrocytoma and oligodendroglioma, no endothelial glycocalyx is observed in (**b**) and (**c**). However, the endothelial glycocalyx is observed in (**e**) and (**f**) with the use of lanthanum nitrate (arrowheads). The arrows indicate the presence of multiple thin rod-shaped glycocalyx (**e**). Thus, SEM with lanthanum staining reveals the endothelial glycocalyx in human glioma vessels. Similar to SEM, no endothelial glycocalyx is observed in (**g–i**), but the endothelial glycocalyx can be observed in (**j–l**), as indicated by the arrowheads. IDH, isocitrate dehydrogenase; SEM, scanning electron microscopy; TEM, transmission electron microscopy

We used electron microscopy to assess the microstructure of the endothelium in gliomas. Lanthanum nitrate was used to visualize the endothelial glycocalyx [21, 22]. Figure 1 shows the tumor vessels of glioblastoma (Case 3), astrocytoma (Case 2), and oligodendroglioma (Case 6) using SEM and TEM. No endothelial glycocalyx was observed in the tumor vessels without lanthanum nitrate (Fig. 1a–c). In contrast, the tumor endothelial glycocalyx was visualized in all three types of gliomas using lanthanum nitrate (Fig. 1d–f). TEM with lanthanum nitrate also revealed an endothelial glycocalyx but not without lanthanum nitrate in the tumor vessels of the glioma (Fig. 1g–l). The other six cases also had endothelial glycocalyx in the tumor vessels. Thus, electron microscopy with lanthanum staining showed the endothelial glycocalyx in human glioma vessels.

### Endothelial glycocalyx may differ in shape in the tumor margin and core

**Fig. 2:**
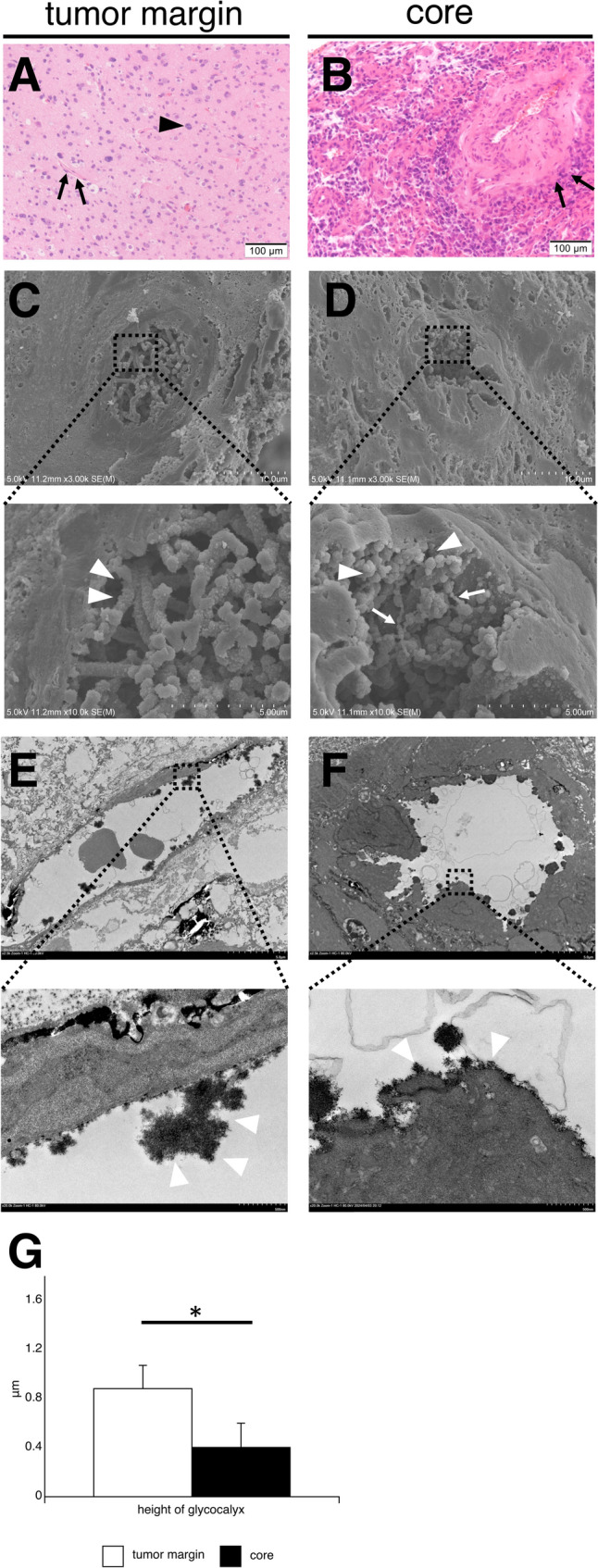
Different microstructure of the endothelial glycocalyx in human glioma cores and the tumor margin. In Gd-MRI, the vasculature of the Gd-enhanced glioma core area is compared with that of the non-Gd-enhanced margins. The upper panel shows HE staining, and the lower panel shows SEM images with lanthanum staining. Representative images of glioblastoma, IDH-wildtype (Case 3). HE staining shows multiple tumor vessels (black arrows) present in the core (**b**), whereas tumor vessels are not as prominent in the tumor margins (**a**). Black arrowheads indicate multinucleated tumor cells. SEM images show a similarly thick rod-like glycocalyx at the tumor margins (**c**) (white arrowheads). In contrast, the tumor endothelium with an irregular lumen has various glycocalyx shapes, such as linear (white arrowheads) and spherical (white arrows) in the cores (**d**). Different three-dimensional microstructures of the endothelial glycocalyx are observed in the tumor margin and core using SEM. TEM images show the endothelial glycocalyx at tumor margin (**e**) and in core (**f**). The height of the endothelial glycocalyx is significantly greater at the tumor margin (*n* = 3) compared to within the core (*n* = 9) (G) (0.88 ± 0.2 µm vs. 0.4 ± 0.19 µm, tumor margin vs. gliomas, *p* < 0.01). Asterisk indicates significant differences between the tumor margin and core. Data are expressed as means ± standard error of the means. Gd-MRI, gadolinium-enhanced magnetic resonance imaging; HE, hematoxylin and eosin; IDH, isocitrate dehydrogenase; SEM, scanning electron microscopy; TEM, transmission electron microscopy

We compared the endothelial glycocalyx in the tumor core (*n* = 9) and margins (*n* = 3; Cases 3, 4, and 8). The gadolinium-enhanced area represented the tumor core and the non-gadolinium-enhanced area represented the tumor margin [23]. The gadolinium-enhanced areas were determined during surgery with navigation system. To compare the microstructure of the endothelial glycocalyx in both, we used SEM with lanthanum nitrate. In three-dimensional observation using SEM, the endothelial glycocalyx in the tumor margins was regular, thick, and rod-shaped (Fig. 2c). In contrast, the tumor core endothelium with an irregular lumen has various glycocalyx shapes, such as linear (white arrowheads) and spherical (white arrows) (Fig. 2d). The height of the endothelial glycocalyx in TEM images was significantly greater at the tumor margin (*n* = 3) compared to within the core (*n* = 9) (G) (0.88 ± 0.2 µm vs. 0.4 ± 0.19 µm, tumor margin vs. core, *p* < 0.01). These results suggest that the microstructure of the endothelial glycocalyx may differ in the tumor margin and core.

### Endothelial glycocalyx components may differ in the gliomas and the tumor margin

**Fig. 3:**
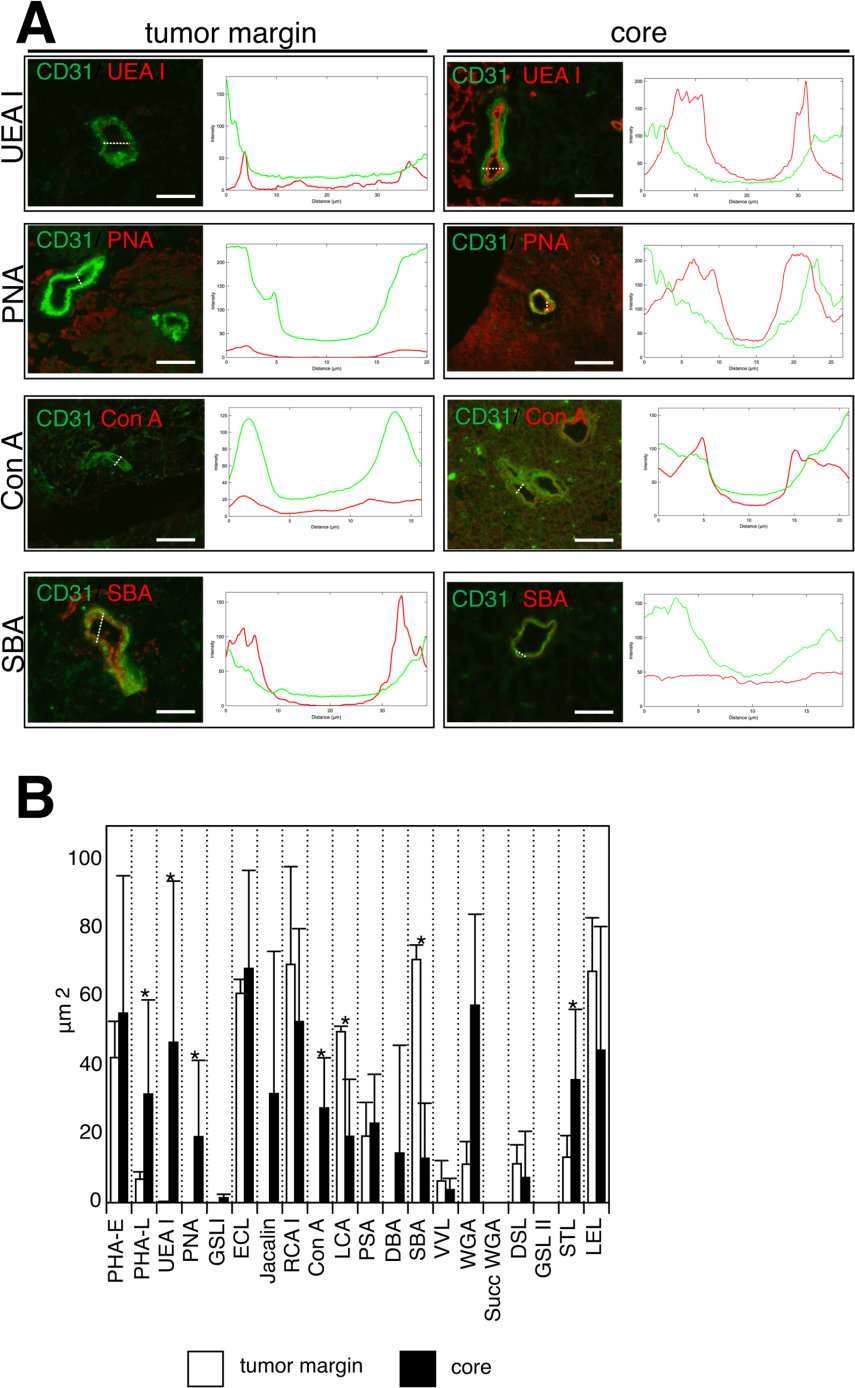
Lectin staining for the endothelial glycocalyx component in the human glioma core and tumor margin. **a** To investigate the differences in the endothelial glycocalyx components, which are the carbohydrate chains, of the tumor margins and cores, specific carbohydrate-binding activity of lectins is used for vessels in the tumor margin and core. Representative lectin staining of the tumor margin and core vessels. Merged images of the endothelial marker CD31 in green, and various lectins in red are shown. The curve graph shows the fluorescence intensity corresponding to the straight lines drawn on the vessels. In UEA I binding to fucose, the vessel lumen did not show high intensity in the tumor margin. However, the core vessel lumen showed high intensity in UEA I. PNA binding to galactose and Con A binding to mannose are not detected in the tumor margin vessel lumen but detected in the core vessel lumen. SBA binding to N-acetylgalactosamine indicated high intensity in the tumor margin lumen. In contrast, the core vessel lumen was not detected in SBA. Further details on the lectins are provided in Table S2. Scale bars = 50 μm. PNA, peanut agglutinin; SBA, soybean agglutinin; UEA, Ulex europaeus agglutinin. **b** The endothelial glycocalyx components of the tumor margin (*n* = 3) and core (*n* = 8) were compared. The areas where the intensity profile showed higher values for the red channel (lectins) than for the green channel (CD31) were calculated in the curve graph of the 20 different types of lectin staining. The area of the vessel lumen is significantly different for the tumor margin and cores in PHA-L (6.9 ± 2.1 µm^2^ vs. 31.7 ± 27.5 µm^2^, tumor margin vs. cores, *p* = 0.02), UEA I (0.3 ± 0.1 μm^2^ vs. 46.8 ± 47.1 μm^2^, tumor margin vs. cores, *p* = 0.02), PNA (0 vs. 19.2 ± 22.3 µm^2^, tumor margin vs. cores, *p* = 0.04), Con A (0 vs. 27.6 ± 14.6 µm^2^, tumor margin vs. cores, *p* = 0.02), LCA (49.9 ± 1.6 µm^2^ vs. 19.3 ± 16.7 µm^2^, tumor margin vs. cores, *p* = 0.02), SBA (71.0 ± 4.2 µm^2^ vs. 12.9 ± 16.1 µm^2^, tumor margin vs. cores, *p* = 0.02), and STL (13.3 ± 6.3 µm^2^ vs. 35.9 ± 20.6 µm^2^, tumor margin vs. cores, *p* = 0.03). These results indicate that the glioma core endothelial glycocalyx may consist of different components from those of the tumor margin. Asterisks indicate significant differences between the tumor margin and core. Data are expressed as the means ± standard error of the means. LCA, Len culinaris lectin; SBA, soybean agglutinin; STL, *Solanum tuberosum* lectin; UEA, Ulex europaeus agglutinin

To compare the endothelial glycocalyx component, we conducted double staining of the frozen sections of gliomas (*n* = 8, except for Case 1) and tumor margin (*n* = 3; Cases 3, 4, and 8) with fluorescently labeled lectins and anti-CD31 antibodies. In this experiment, we used OCT-embedded frozen tissues because they undergo minimal processing and preserve the natural distribution of glycans, which are naturally hydrated [24]. Details of the lectins used are summarized in Table S2. Figure 3a shows representative lectin staining of the tumor margin and core vessels. Merged images of the endothelial marker CD31 in green and various lectins in red are shown. The graph shows the fluorescence intensity corresponding to the straight lines drawn on the vessels. For Ulex europaeus agglutinin I (UEA I) that binds to fucose, the vessel lumen did not show a high intensity in the tumor margin. However, the core vessel lumen showed a high intensity for UEA I staining. Peanut agglutinin (PNA) binding to galactose and concanavalin A (Con A) binding to mannose were not detected in the tumor margin vessel lumen but were observed in the core vessel lumen. Soybean agglutinin (SBA) binding to *N*-acetylgalactosamine indicated high intensity in the tumor margin lumen. In contrast, the core vessel lumen was not detected in SBA. The areas of the region where the red signal intensity of the red channel was higher than that of the green channel were evaluated for the 20 different lectins. Lectin staining in the vessel lumen revealed the endothelial glycocalyx components. There were significant differences between the tumor margin and core in Phaseolus vulgaris leucoagglutinin (6.9 ± 2.1 µm^2^ vs. 31.7 ± 27.5 µm^2^, tumor margin vs. core, *p* = 0.02), UEA I (0.3 ± 0.1 μm^2^ vs. 46.8 ± 47.1 μm^2^, tumor margin vs. core, *p* = 0.02), PNA (0 vs. 19.2 ± 22.3 μm^2^, tumor margin vs. core, *p* = 0.04), Con A (0 vs. 27.6 ± 14.6 μm^2^, tumor margin vs. core, *p* = 0.02), Len culinaris lectin (49.9 ± 1.6 μm^2^ vs. 19.3 ± 16.7 μm^2^, tumor margin vs. core, *p* = 0.02), SBA (71.0 ± 4.2 μm^2^ vs. 12.9 ± 16.1 μm^2^, tumor margin vs. core, *p* = 0.02), and *Solanum tuberosum* lectin (13.3 ± 6.3 μm^2^ vs. 35.9 ± 20.6 μm^2^, tumor margin vs. core, *p* = 0.03) (Fig. 3B). These results demonstrate significant differences in the endothelial glycocalyx components of the tumor margin and core (Table S3).

## Discussion

In this study, the microstructures of tumor vessels in human gliomas were visualized using SEM and TEM. The results revealed the presence of an endothelial glycocalyx in the tumor vessels of gliomas. Furthermore, the microstructures of the endothelial glycocalyx differed in tumor margins and cores. In addition, staining with 20 different lectins showed differences in the endothelial glycocalyx components of tumor margins and cores.

Several studies on the microstructure of brain tumor vessels have used TEM [6, 7, 25]. In this study, SEM was used for the first time to observe human brain tumors. SEM is excellent for observing tissue surfaces and can detect three-dimensional microstructures. Using SEM, we identified the fine structure of the endothelial glycocalyx in the tumor vessels of human gliomas. Most studies investigating the structure and characteristics of the glycocalyx present on the surface of endothelial and tumor cells have been conducted using animal models, such as mice and rats, or cultured human cells [16]. However, these findings do not accurately represent the characteristics and appearance of the human glycocalyx. Therefore, we directly visualized and histologically evaluated the human cerebral endothelial glycocalyx. The images in this study are likely to have significant implications for future glioma chemotherapy, particularly in areas such as drug delivery.

The endothelial glycocalyx is involved in tumor growth, invasion, and metastasis in animal models [13, 26, 27]. However, these findings are not characteristic of the human endothelial glycocalyx [17, 28]. Studies demonstrating the role of the endothelial glycocalyx in human malignancies are limited [22]. This study not only revealed the presence of the endothelial glycocalyx in human gliomas but also indicated that its microstructure may differ in tumor margins and cores. Further studies on the previously unknown endothelial glycocalyx in human gliomas may lead to therapies targeting tumor blood vessels. Therefore, there is an urgent need to clarify the specific function of the endothelial glycocalyx in human gliomas and how it is involved in glioma survival.

It has been reported that BBB changes in an in vitro model induced by glioma-conditioned media are associated with increased glycan expression such as WGA, STL, UEA I, DBA, and PNA, similar to the current findings [29]. Changes in the glycocalyx on the tumor cell membrane have been shown to promote glioma progression, tumor cell adhesion and migration, and the stiffening of the tumor extracellular matrix [30]. From our results, changes in the components of the endothelial glycocalyx may affect vascular permeability and tumor extracellular matrix, which may be related to tumor growth, invasion, and treatment resistance.

Biological material itself appears with poor contrast in electron microscopy, owing to its composition mostly of light elements. Classical staining agents like osmium tetroxide, uranyl acetate, and lead citrate preserve and/or stain cellular structures such as membranes, cytoplasm, and organelles well for electron microscopy. However, proteoglycans, a main component of the endothelial glycocalyx, show no or only poor contrast with these conventional staining agents. These can be visualized by electron microscopy only by additional staining with heavy metal ions such as copper, ruthenium, or lanthanum [11]. In past reports, osmium was used to observe the glycocalyx of cell membranes, but sufficient contrast was not obtained [14]. Based on the results of this study, we believe that lanthanum is suitable for observing the endothelial glycocalyx in human brain tumor vessels.

This study had several limitations. First, the sample size was small. Second, it remains unclear how the tumor endothelial glycocalyx acts as a blood–tumor barrier. To overcome these limitations, it is necessary to elucidate the function of the endothelial glycocalyx in human gliomas. This study is the first step in this process of identifying the tumor endothelial glycocalyx in human gliomas and examining its microstructure. We fully acknowledge that the number of cases in this paper is relatively small. Although we believe our current findings provide valuable insights into the endothelial glycocalyx in gliomas, further investigation with a larger cohort is necessary to strengthen and expand upon these observations.

## Conclusion

The present study revealed the microstructure of endothelial glycocalyx in the tumor vessels of human gliomas. The endothelial glycocalyx not only differs in shape but also in composition between the tumor margin and core. The results of this study may provide a basis for the future development of therapies targeting tumor blood vessels. Further studies are expected to provide a more detailed analysis of the tumor endothelial glycocalyx.

## Supplementary Information

Below is the link to the electronic supplementary material.

Supplementary file 1 (DOCX 261309 KB)

## Data Availability

Data are available upon reasonable request.
