# Supplementary material for: Visualizing the endothelial glycocalyx in human glioma vasculature
Source: Brain Tumor Pathol. 2025 Mar 4;42(2):33–42. doi: 10.1007/s10014-025-00498-z (PMC11993481; doi:10.1007/s10014-025-00498-z)
Supplement: Supplementary file 1 — (DOCX 261309 KB) [file 10014_2025_498_MOESM1_ESM.docx]

**Fig. S1** Tumor vessels of human gliomas are observed in the gadolinium-enhanced MRI images.

Representative images of gliomas: (A–C) Glioblastoma, IDH-wildtype (case 3); (D–F) Astrocytoma, IDH-mutant, grade4 (case 2); and (G–I) Oligodendroglioma, IDH-mutant and 1p/19q-codeleted, grade 3 (case 6). HE staining (B, E, H) and CD31 immunostaining (C, F, I) of the gadolinium-enhanced areas in MRI images are shown. Glioblastoma shows typical ring enhancement (A) and increased endothelium in the tumor vessels (B). The tumor vessels are strongly positive for CD31 (C). Astrocytomas (D–F) and oligodendrogliomas (G–I) show CD31-positive tumor vessels in a slightly contrasted area. The tumor vessels of gliomas are commonly positive for CD31, an endothelial marker. Thus, tumor vessels are observed in the gadolinium-enhanced areas in all three types of gliomas. HE, hematoxylin and eosin; IDH, isocitrate dehydrogenase; MRI, magnetic resonance imaging

**Tables**

Table S1. Patient characteristics

| **Case number** | **Diagnosis** | **Sex** | **Age, years** | **New or Recurrence** | **Treatment** |
| --- | --- | --- | --- | --- | --- |
| 1 | Astrocytoma, IDH-mutant, grade 4 | F | 54 | recurrence | TMZ+RT |
| 2 | Astrocytoma, IDH-mutant, grade 4 | F | 50 | new |  |
| 3 | Glioblastoma, IDH-wildtype | M | 76 | new |  |
| 4 | Glioblastoma, IDH-wildtype | M | 60 | new |  |
| 5 | Glioblastoma, IDH-wildtype | M | 82 | new |  |
| 6 | Oligodendroglioma, IDH-mutant and 1p/19q-codeleted, grade 3 | F | 61 | recurrence | TMZ, PAV |
| 7 | Astrocytoma, IDH-mutant, grade 4 | M | 26 | recurrence | TMZ+RT, TTFs |
| 8 | Glioblastoma, IDH-wildtype | M | 80 | new |  |
| 9 | Glioblastoma, IDH-wildtype | F | 79 | new |  |

IDH, isocitrate dehydrogenase; F, female; M, male; TMZ, temozolomide; RT, radiation therapy; PAV, procarbazine, ACNU, and vincristine; TTFs, Tumor Treating Fields.

Table S2. Summary of the 20 lectins used in this study

| **Primary Sugar Specificity** | **Source of Lectin** | **Common Abbreviation** |
| --- | --- | --- |
| Complex structures | Phaseolus vulgaris Erythroagglutinin | PHA-E |
| Complex structures | Phaseolus vulgaris Leucoagglutinin | PHA-L |
| Fucose | Ulex Europaeus agglutinin I | UEA I |
| Galactose | Peanut Agglutinin | PNA |
| Galactose | Griffonia simplicifolia lectin I | GSLI |
| Galactose | Erythrina cristagalli lectin | ECL |
| Galactose | Jacalin | Jacalin |
| Galactose | Ricinus communis agglutinin I | RCA I |
| Mannose | Concanavalin A | Con A |
| Mannose | Len culinaris lectin | LCA |
| Mannose | Pisum sativum agglutinin | PSA |
| N-Acetylgalactosamine | Dolichos biflorus agglutinin | DBA |
| N-Acetylgalactosamine | Soybean agglutinin | SBA |
| N-Acetylgalactosamine | Vicia villosa lectin | VVL |
| N-Acetylglucosamine | Wheat Germ agglutinin | WGA |
| N-Acetylglucosamine | Wheat Germ agglutinin, succinylated | Succ WGA |
| N-Acetylglucosamine | Datura stramonium lectin | DSL |
| N-Acetylglucosamine | Griffonia simplicifolia lectin Il | GSL II |
| N-Acetylglucosamine | Solanum tuberosum lectin | STL |
| N-AcetyIglucosamine | Lycopersicon esculentum lectin | LEL |

Table S3. Summary of lectins that differed between the tumor margin and core

| **Common Abbreviation** | **Primary Sugar Specificity** | **Tumor Margin** | **Core** |
| --- | --- | --- | --- |
| PHA-L | Complex structures |  | + |
| UEA I | Fucose |  | + |
| PNA | Galactose |  | + |
| Con A | Mannose |  | + |
| LCA | Mannose | + |  |
| SBA | N-Acetylgalactosamine | + |  |
| STL | N-Acetylglucosamine |  | + |

+ indicates significantly higher expression than the other.
